# The Effectiveness of Nursing Rehabilitation Interventions on Self-Care for Older Adults with Respiratory Disorders: A Systematic Review with Meta-Analysis

**DOI:** 10.3390/ijerph20146422

**Published:** 2023-07-20

**Authors:** Rita Ribeiro, Henrique Oliveira, Margarida Goes, Cátia Gonçalves, Ana Dias, César Fonseca

**Affiliations:** 1 Nursing Department, University of Évora, 7000-801 Évora, Portugal; ritiribeiro@hotmail.com (R.R.); mgoes@uevora.pt (M.G.); catia.goncalves84@gmail.com (C.G.); anadias@uevora.pt (A.D.); cfonseca@uevora.pt (C.F.); 2 Institute of Telecommunications, 3840-193 Aveiro, Portugal; 3 Polytechnic Institute of Beja, 7800-295 Beja, Portugal; 4 Comprehensive Health Research Centre (CHRC), University of Évora, 7000-801 Évora, Portugal

**Keywords:** self-care, respiratory rehabilitation, nursing interventions, health outcomes

## Abstract

**Background**: This research work aimed to summarize the rehabilitation nursing interventions published in the scientific literature that most contribute to effective adherence to self-care in older adults with respiratory diseases. **Methods**: A systematic literature review with meta-analysis was conducted by searching the EBSCOhost platform (CINAHL Complete, MEDLINE Complete, Cochrane, and MedicLatina) using the PRISMA methodology. Five articles were selected for final analysis. Meta-analysis was carried out using Comprehensive Meta-Analysis (CMA) software, and the results were presented in a forest plot. **Results**: Thirty-one self-promoting rehabilitation nursing interventions were identified, with the most effective being those related to the assessment of progress in physical capacity/activity tolerance (functional status category/domain) and the assessment of the increase in health-related quality of life (health-related quality of life category/domain). **Conclusions**: Rehabilitation nursing interventions such as self-management programs led by nurses, community-based and home-based rehabilitation programs, and inspiratory muscle training can effectively reduce and enable the effective control of symptoms associated with respiratory disorders, boosting older adults’ empowerment to engage in self-care.

## 1. Introduction

Significant demographic changes are underway globally, with the world’s population aging rapidly. The pace of aging has accelerated in the past decade and is expected to accelerate even faster in the next three decades. The increase in life expectancy and the decrease in birth rates have contributed to this trend. However, even though people live longer, the extra years of life are often unhealthy. The reduction in the number of years of a healthy life, which significantly impacts individuals aged 65 and older, is related to the high prevalence of multiple and complex chronic disorders (multimorbidity) that cause disabilities and dependencies. As people age, their respiratory systems undergo natural changes that can increase the risk of respiratory disorders. Some of these changes include decreased elasticity of the lung tissue, weakened respiratory muscles, and reduced lung capacity [1]. These changes can make breathing more difficult for older adults, leading to a higher risk of respiratory disorder prevalence (chronic and infectious conditions) [2]. Other factors such as smoking, air pollution exposure, and underlying medical conditions such as heart disease can aggravate these changes and increase the risk of respiratory disorders in older adults [3,4].

A systematic analysis regarding the “Global Burden of Disease Study 2019” reported that chronic disorders accounted for nine of the top ten causes of death worldwide, with respiratory disorders considered one of the most prevalent non-communicable diseases [5]. In 2017, chronic respiratory disorders were the third leading cause of all deaths (70% (95% UI 6.8–7.2)) [6]. The two most common chronic respiratory disorders are chronic obstructive pulmonary disease (COPD) and asthma, followed by occupational lung diseases and pulmonary hypertension [7]. Chronic respiratory disorders can be categorized as obstructive or restrictive. These respiratory disorders, such as COPD and asthma, restrict airflow and impair lung function. In older adults, the prevalence of these conditions increases, leading to a higher risk of exacerbation, hospitalization, and mortality. Furthermore, overlap syndromes, such as the coexistence of obstructive lung disease and sleep apnea syndrome, further complicate the health of older adults. The interplay of these conditions exacerbates symptoms, impairs quality of life, and necessitates comprehensive management strategies for improved outcomes in this vulnerable population [8,9].

Concerning infectious respiratory disorders, they are caused by pathogens and are acute, requiring antimicrobial or antiviral treatments, with the great majority of death caused by lower respiratory infections—predominantly pneumonia [10]. Rehabilitation nursing interventions (RNIs) for infectious diseases focus on airway clearance and breathing efficiency [11,12].

Respiratory disorders, whether resulting from chronic or infectious conditions, often cause airflow limitation, causing the symptom of shortness of breath, called dyspnea, which reduces exercise capacity, limits functionality, and impairs the performance of Activities of Daily Living (ADL). RNIs play a crucial role in assisting patients presenting these symptoms. These interventions include educating patients on breathing techniques, assisting with physical activity planning and exercise training, implementing strategies to conserve energy, and offering emotional support. Through these interventions, rehabilitation nursing aims to improve patients’ respiratory function, ability to carry out daily activities, and overall quality of life [8,13,14].

To assess the severity and progression of respiratory disorders, nursing care-sensitive indicators (outcome measurements) are used to ascertain whether patients respond positively to nursing interventions and help determine whether changes in care are needed. They are crucial to providing effective RNIs [15]. Some common sensitive indicators include respiratory rate, oxygen saturation levels, lung auscultation findings, cough frequency, and sputum production. These indicators help healthcare professionals monitor the respiratory status of patients, identify any deterioration or improvement, and guide appropriate interventions. By closely monitoring these indicators, rehabilitation nurses can tailor their care plans, administer respiratory treatments, provide appropriate breathing exercises, and implement necessary respiratory support, ultimately improving patients’ outcomes and quality of life [16,17].

Long-term nursing care may be required depending on the severity and progression of respiratory disorders, which are common among older adults and can significantly impact their quality of life. Respiratory disorders such as COPD, asthma, and pulmonary fibrosis can be chronic and may worsen over time, leading to a decline in lung function and overall health [8,9]. In some cases, older adults with respiratory disorders may require assistance with daily activities such as bathing, dressing, and using the bathroom. They may also require oxygen therapy or other treatments to help manage their symptoms. Thus, nursing care can provide a safe and supportive environment for older adults with respiratory disorders, helping them to manage their health conditions and prevent their worsening [18].

In recent decades, RNIs has gained significant recognition as a powerful approach to addressing respiratory disorders, enhancing functional abilities, optimizing health outcomes, and promoting self-care skills and overall quality of life [19,20]. By adopting self-care measures, individuals can acquire knowledge and skills to manage their health conditions appropriately, reducing dependence on costly medical care and minimizing disease-related complications [21,22]. Notably, older adults with respiratory disorders stand to benefit immensely from engaging in self-care practices. These proactive measures enable them to actively manage their symptoms, enhance their overall health and well-being, prevent further deterioration of their health status, and preserve their independence in daily living activities [23]. Self-care practices for older adults with respiratory disorders may include [24,25,26,27]: (i) quitting smoking or avoiding exposure to secondhand smoke; (ii) performing regular exercise to maintain cardiovascular health and lung function; (iii) eating a healthy diet to maintain a healthy weight and reduce inflammation; (iv) practicing good hygiene to prevent respiratory infections; (v) avoiding exposure to environmental irritants, such as pollution or strong odors; (vi) managing stress through relaxation techniques, such as deep breathing or meditation; (vii) taking prescribed medications as directed by a healthcare provider.

Rehabilitation nursing (RN) can play the following crucial roles in promoting self-care among older adults with respiratory disorders [28,29,30,31]: (i) providing education to patients about their conditions and how to manage them through self-care practices, thus including information about diet, exercise, and medication management, among other aspects of care; (ii) working with patients to set goals for their recovery and promote self-care practices that align with those goals, helping patients to feel empowered and motivated to take an active role in their own care; (iii) assessing patients’ abilities and limitations and developing individualized care plans that promote self-care, involving identifying areas where patients need assistance or support, as well as areas where patients can take an active role in their own care; (iv) providing emotional support and encouragement to patients as they navigate the challenges of recovery, thus helping patients to feel more confident in their ability to manage their own care and engage in self-care practices.

Determining the most effective RNIs for older adults with respiratory disorders can be particularly challenging due to the extensive range of rehabilitation options available. Therefore, a systematic literature review with meta-analysis (SLR-MA) was conducted to identify, select, and critically evaluate relevant studies on the topic, helping to identify the most effective interventions for older adults suffering from respiratory disorders and areas where further research is needed. The primary objective of this SLR-MA is to highlight the significance of RNIs tailored explicitly to individuals suffering from respiratory disorders, particularly older adults. By consolidating the findings from various studies, this review aims to present a concise and impartial summary of the evidence, enabling healthcare professionals and policymakers to make well-informed choices regarding the most suitable interventions for this specific group of patients. Furthermore, it serves as a roadmap for future research endeavors, ensuring that crucial inquiries in this field are adequately addressed and prioritized. In addition, this SLR-MA also aims to identify the self-care-promoting RNIs that lead to the most significant impact on the various domains of patient well-being. Understanding the role of self-care activities within the analyzed RNIs is crucial to providing comprehensive and evidence-based insight into approaches that may effectively promote disease self-management and improve individuals’ health.

## 2. Materials and Methods

As a methodological approach, the following steps were used to conduct this SLR-MA, whose protocol was registered in PROSPERO [32], with registration number: CRD42022354794: (i) definition of the main question or objective (research question), helping to focus the research process and to determine the scope of the review; (ii) specification of the criteria established to determine which studies will be included or excluded from the review (exclusion and inclusion criteria); (iii) identification of the descriptors or search terms (keywords, subject headings, or specific phrases that relate to the review topic) that are relevant to the research question and introducing them into the web platform that allows for the searching of multiple databases; (iv) searching the various databases selected using the descriptors identified in order to retrieve relevant studies; (v) selection of the studies from the databases by reading the titles and abstracts of the articles in order to assess their relevance to the research question and inclusion/exclusion criteria; (vi) detailed evaluation of the selected articles, involving reading the full text of each article to assess its quality, methodology, and relevance to the research question, a task that was performed by two independent reviewers to ensure consistency; (vii) analysis of the collected data, aiming to extract relevant information from each article, such as study characteristics, methodology, results, and conclusions, in order to synthesize and analyze them to allow for the identification of patterns, trends, and relationships among the included studies [33]. Moreover, this systematic review was conducted and reported in accordance with the Preferred Reporting Items for Systematic Reviews and Meta-Analyses (PRISMA) Statement [34].

A brief meta-analysis procedure was also conducted, mainly: (i) to systematically review and analyze the selected literature on the topic by synthesizing the findings of multiple studies, thus providing a broader and more comprehensive understanding of the subject; (ii) to determine the statistical significance of the findings by combining sample sizes and effect sizes (this represents the magnitude and direction of the treatment effect or relationship under investigation) from multiple studies; (iii) to provide a quantitative estimate of the overall effect size magnitude of the multiple studies selected for analysis [35]. The software used to perform the meta-analysis was Comprehensive Meta-Analysis (CMA), version 4, from Biostat Inc., Englewood, NJ, USA [36]. Regarding the statistical model used, a fixed-effects (singular) model was employed for the analysis [37], and the studies included in the analysis were all drawn from the same population and are identical to each other in all material respects. The results of this analysis will be used to make an inference on this population only and will not be generalized to any other populations. The following statistics were calculated for each study [35,36]: (i) standard differences in means; (ii) standard error of the mean; (iii) variance; (iv) the lower and upper limits of the confidence interval of the mean at the level of 95%; (v) *z*-value; and (vi) *p*-value. A forest plot was also depicted using the same software, which is a graphical representation generally used to present the results of multiple studies, thus providing a visual summary of the treatment effect sizes and confidence intervals of the means for each study, as well as the overall combined effect estimate [36]. The Q-statistic (Q-test for heterogeneity) was also calculated, which provides a test of the null hypothesis that all studies in the analysis share a common effect size. If all studies shared the same true effect size, the expected value of Q would be equal to the degrees of freedom (the number of studies minus 1) [35].

The PI(C)O methodology was the structured approach used in this SLR-MA to formulate the research questions and guide the searches, where PI(C)O stands for *population* (the specific group or type of individual that the research question aims to investigate), *intervention* (the particular treatment, exposure, or diagnostic test that the research question focuses on), *comparison/context/condition* (optional component that identifies the alternative or comparison intervention, treatment, or condition that will be compared with the intervention of interest), and *outcome* (the specific results or effects that are of interest in the research question), with each component representing a key aspect that needs to be clearly defined to ensure a focused and comprehensive review [38].

The following research question was formulated according to the PICO concept: “What are the sensitive indicators of RN (*outcome*) obtained through self-care-interventions (*intervention*) in individuals aged 65 years or older with respiratory disorders (*population*)?”.

Once the PICO question had been formulated, data on the subjects under study were collected during July 2022 using the EBSCOhost platform, and we later selected the following available databases: CINAHL Complete, MEDLINE Complete, Nursing & Allied Health, Cochrane Central Register of Controlled Trials, Cochrane Database of Systematic Reviews, Cochrane Methodology Register, Cochrane Clinical Answers, Library, Information Science & Technology Abstracts, and MedicLatina. The health sciences descriptors used in the search were validated using the “Descritores em Ciências da Saúde”—DeCS (Health Science Descriptors [39]) versus ”Medical Subject Headings” (MeSH [40]), namely: “nursing”; “nursing interventions”; “nursing care”; “respiratory rehabilitation”; “self-care promotion”; “education”; “older adults”; “respiratory disorders”. These descriptors were organized using the Boolean operators OR and AND, in the following arrangement:

(nursing OR nursing interventions OR nursing care) AND (respiratory rehabilitation) AND (self-care promotion OR education) AND (older adults) AND (respiratory disorders).

To impose limits on the research carried out, the following inclusion criteria were chosen: (i) studies published between January 2018 and July 2022; (ii) full-text availability; (iii) those written in the English and Portuguese languages; and (iv) those involving only participants aged 65 years or older. Duplicate articles obtained during the search were excluded, as were those whose descriptors were not correlated with the study objective, despite appearing in the titles of the articles or their abstracts. Studies with ambiguous methodologies were also excluded.

For the initial screening of articles, all titles and abstracts were read to identify those that seemed relevant to the present SLR-MA. If the title and abstract were interesting or inconclusive, the article was read in full to minimize the loss of information valuable to the present SLR-MA. If the article was interesting, it was included in this study.

**Figure 1:**
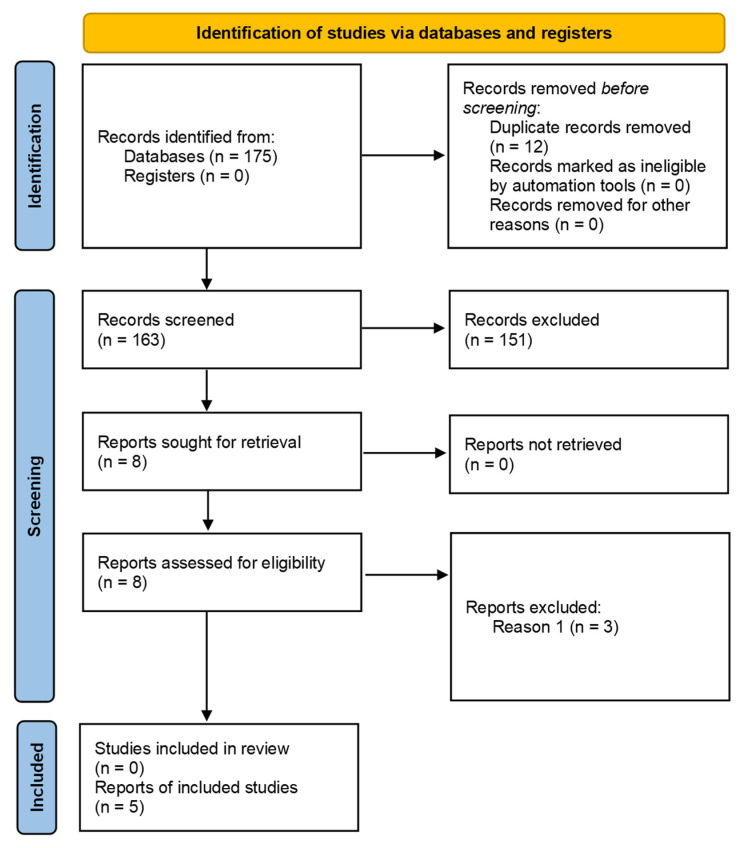
PRISMA flow diagram representing the research pathway.

The search conducted in the databases mentioned above resulted in 175 articles. After eliminating 12 articles because they were considered duplicates, 163 were retrieved, from which the respective titles and abstracts were extracted for analysis. After reading the titles/abstracts, following the criteria mentioned in the previous paragraph, 155 articles were eliminated since they did not fit the study’s subject. After that, 8 articles were selected for full-text analysis. Two independent reviewers reviewed all 8 articles regarding their methodological quality and evidence levels to ensure the consistency of the analyses, using the Critical Appraisal Tools of the Joanna Briggs Institute (JBI) [41,42]. Four articles (Chung et al. [8], Martin-Sanchez et al. [13], Wang et al. [9], Collins et al. [43]) had methodological quality values greater than 50% and presented the following level of evidence: *1.c—Randomized Controlled Trial*, considered the gold standard in clinical research because is provides the highest level of evidence for evaluating the effectiveness of interventions. However, a fifth study (Tsang et al. [14]) was also included in this SLR-MA, despite having the level of evidence *3e*, because it met the inclusion criteria set for the review, and no further higher-level studies were available. Although this study may have limitations concerning controlling variables and potential biases, it provides valuable information for the review, fills knowledge gaps, and explores different aspects of the research question. Thus, only 5 articles were included in the present SLR-MA. The flow chart representing the research pathway is shown in Figure 1, using the PRISMA flow diagram [34].

The five articles were carefully screened to identify the self-care activities conducted in the context of RNI. Such self-care activities were then listed in descending order of treatment effect size, as determined in the forest plot.

Finally, based on the work proposed by Doran and Pringle [44], the following framework of patient outcome indicators was used, to systematize the nursing interventions that had a more significant impact on the various domains of the well-being of patients with respiratory disorders, following the recommendations of the RN specialty nurses’ board of the Portuguese Order of Nurses [45], namely: (i) *functional status*, which includes outcomes related to the physical and emotional functioning of the patient, such as improved mobility, ability to perform daily activities, and relief of physical and emotional symptoms, among others; (ii) *symptom control*, which encompasses outcomes related to the effective management of symptoms and discomfort experienced by patients, such as pain relief, anxiety control, and the reduction in fatigue, among others; (iii) *self-care capacity*, which refers to outcomes related to patients’ ability to care for themselves and perform self-care activities, such as personal hygiene skills, proper medication administration, and diet management, among others; (iv) *quality of life*, which involves outcomes related to the overall perception of the well-being and life satisfaction of the patient, considering physical, emotional, social, and environmental aspects; (v) *health promotion activities*, which includes outcomes related to patients’ engagement in health promotion activities, such as adherence to prevention programs, participation in physical activities, and healthy eating habits, among others; (vi) *healthcare utilization*, which encompasses outcomes related to the appropriate utilization of healthcare services by the patient, such as the frequency of medical appointments, adherence to prescribed treatments, and utilization of emergency services, among others; and (vii) *patient satisfaction*, which refers to outcomes related to patients’ perceptions of and satisfaction with the care received, considering communication with the nursing team, the quality of the care environment, and access to services, among others. Based on the proposed indicators, this adapted classification framework allows for a comprehensive analysis of patient outcomes sensitive to nursing care in the context of RN.

## 3. Results

The five articles were read in full to reach the proposed objectives, and their content was analyzed. The characteristics and main results are summarized in Table 1, in ascending chronological order of publication. Regarding the sensitive indicators of RNIs in older adults with respiratory problems, those found in the mentioned references are listed in Table A1 in Appendix A to reduce the complexity of Table 1.

A brief meta-analysis was also conducted based on the functioning variable, which was replicated in the four studies eligible for this review. The 6-min walking test (6MWT) was used as an instrument to assess the functioning variable, after the implementation of the Rehabilitation Programs (RP) in the studies of Chung et al. [8] (breathing exercise training (BTE) and inspiratory muscle training (IMT) groups), Martin-Sanchez et al. [13] (inspiratory muscle training with high intensity (IMT High) and inspiratory muscle training with low intensity (IMT Low)), Wang et al. [9] and Tsang et al. [14] (the Community-Based Rehabilitation Program (CBRP) and the Home-Based Rehabilitation Program (HBRP)), whose results are listed in Table 2, where the effect size index is the standardized difference in means between the control and intervention groups regarding the 6MWT variable. The common effect size for these studies is 0.682 with a 95% confidence interval of 0.536 to 0.827. The treatment effect size in this population could fall anywhere in this interval. The *Z*-value tests the null hypothesis that the common effect size is zero. The *Z*-value is 9.186, with *p* < 0.001. Using a criterion alpha of 0.050 [35], we reject the null hypothesis and conclude that in this population, the treatment effect size is not precisely zero. The Q-value is 18.807 with six degrees of freedom and *p* = 0.005. Using a criterion alpha of 0.100 [35], we can reject the null hypothesis that the true effect size is the same in all these studies.

**Figure 2:**
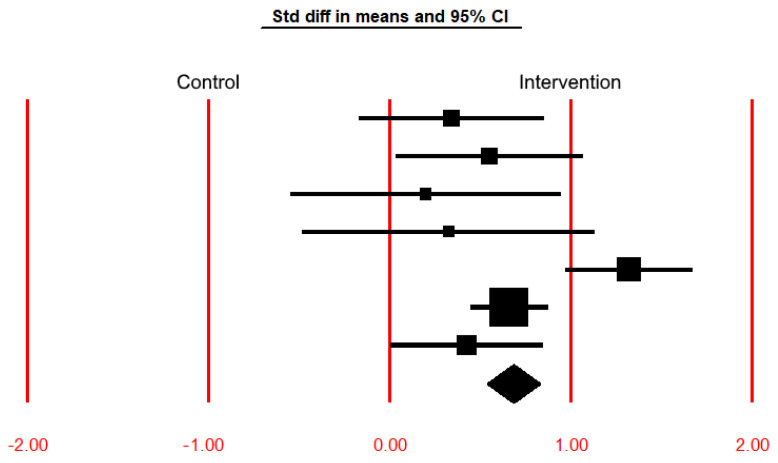
Graphical representation (forest plot) of the treatment effect sizes (the effect size index is the standardized difference in means) and 95% confidence intervals of each study for the results shown in Table 2. The diamond represents the point estimate of the averaged studies.

The results in Table 2 are represented graphically in the forest plot shown in Figure 2. It includes a vertical line indicating the null treatment effect at “0.00” (e.g., no difference between groups or no association). The other vertical lines at “1.00” and “2.00” represent the standardized difference in means of 1.0 and 2.0, respectively, corresponding to a positive treatment effect. In contrast, the vertical lines at “−1.00” and “−2.00” represent the standardized difference in means of −1.0 and −2.0, corresponding to a negative treatment effect. Squares represent individual study estimates, where the size of each square or circle represents the weight of the study in the meta-analysis and is proportional to the sample size or precision of the estimate. The greater the distance of the square relative to the right of the vertical line at “0.0”, the greater the positive effect of the treatment. On the contrary, the greater the distance of the square relative to the left of the vertical line at “0.0”, the greater the negative effect of the treatment. The horizontal line extending from each square represents the confidence interval of the treatment effect estimate, indicating the range of plausible values (its left and right lengths are the lower and upper bounds shown in Table 2). For the treatment effect to be considered statistically significant (either positive or negative), the respective horizontal line cannot cross the vertical line at “0.00”. The effects of RNIs are considered statistically significant for the following studies: (i) Chung et al. [8]—IMT; (ii) Wang et al. [9]; (iii) Tsang et al. [14]—CBRP; and (iv) Tsang et al. [14]—HBRP, since the *p*-value is less than 0.05 (the lower limit of the 95% confidence interval is greater than 0.000—see Table 2—with the respective horizontal lines of the forest plot not crossing the reference line 0.000 to its negative side—see Figure 2).

## 4. Discussion

This SLR-MA aimed to systematize valuable information on RNIs for older adults with respiratory disorders through a comprehensive analysis of five relevant studies. As the main findings report, a self-management program led by nurses successfully reduced hospital readmissions and emergency department visits, and enhanced exercise capacity, health-related quality of life, and patient satisfaction among individuals diagnosed with COPD. Community-based and home-based rehabilitation programs enhanced patients’ exercise tolerance and knowledge of the disease. Community-based rehabilitation programs mainly addressed patients’ mental and psychosocial needs, yielding more significant advantages, while home-based rehabilitation programs primarily improved patients’ physical function without significantly affecting their mental health or health-related quality of life. Inspiratory muscle training shows greater efficacy compared to breathing exercise intervention when it comes to improving respiratory muscle strength, and it may serve as a viable alternative to conventional breathing exercises, potentially producing positive results in middle-aged and elderly patients with asthma. By identifying these primary results, it was possible to realize that all studies’ results converge toward the importance of continuous monitoring, family involvement, peer support, and health education in promoting self-care in older adults with chronic diseases. These factors are associated with patient empowerment, increased health literacy, emotional support, and improved self-care outcomes, reinforcing the belief that integrated approaches are essential to promoting effective self-care.

The studies carried out by Wang et al. [9] and Chung et al. [8] highlighted the benefits of post-pulmonary rehabilitation (PR) follow-up in promoting patients’ self-care. These follow-up interventions included structured monitoring and telephone-based health education, empowering patients and caregivers and leading to increased health literacy and social support. Thus, active and continuous monitoring was proven to empower patients to engage in self-care. Both studies also underlined the integration of family and caregivers in the self-care process. In a study carried out by Tsang et al. [14], the authors support these results by affirming that family education sessions were associated with reducing depressive symptoms in patients. Family members’ participation in these sessions was also related to increased patient knowledge about the disease, which reveals how family involvement can be a crucial factor in self-care by providing emotional support, helping with physical care, and contributing to disease education and comprehension. Following the research developed by López-Liria et al. [18], the authors also corroborate these findings and emphasize the importance of the relationship between patients and families. They propose a family-centered approach, identifying the patient’ and families’ specific needs to target care more effectively. This personalized approach makes it possible to evaluate care outcomes and adapt care plans according to patients’ real needs.

Another factor mentioned in the discussion is peer support, which was made clear in the study by Tsang et al. [18]. Sharing experiences among patients with the same disorder seemed to reduce symptoms of anxiety, depression, and loneliness, promoting a sense of empathy and psychological support. This type of mutual support among peers can be a valuable source of emotional support and promotion of psychological and mental well-being. The mentioned studies observed the importance of continuous monitoring, family involvement, and peer support in promoting self-care in chronic diseases. Based on our screening of the scientific evidence produced in the national context (in Portugal), several authors have addressed this subject and corroborate these findings by stating that these interventions contribute to the empowerment of patients, increase health literacy and emotional support, and provide a better understanding of the disease, resulting in better self-care outcomes and overall well-being [46,47,48,49,50].

Following the framework of patient outcome indicators proposed by Doran and Pringle [44], as mentioned in the Section 2, used to systematize the nursing interventions found in this research, *functional status* encloses RNIs that aim to promote a person’s functionality, primarily through respiratory functional re-education (RFR) programs and exercising. The studies carried out by Wang et al. [9], Chung et al. [8], Martin-Sanchez et al. [13], Collins et al. [43], and Tsang et al. [14] reported interventions such as the assessment of progress in physical ability/activity tolerance as ways to promote self-care. These studies showed an increase in 6-min walk test (6MWT) scores after the implementation of pulmonary rehabilitation, resulting in an average increase in patients’ functional capacity. The evaluation of pulmonary function [8,13,14,43] and functional capacity assessment were relevant in the cited papers. These assessments made it possible to address patients’ parameters in an individually tailored way, with interventions targeted at the previous diagnosis, improving their functional status. The study carried out by Uslu and Canbolat [51] corroborates these findings. The authors Wang et al. [9], López-Liria et al. [18], Collins et al. [43], Tsang et al. [14], and Martin-Sanchez et al. [13] highlighted the importance of exercising in improving patients’ functional capacity and physical condition. They also corroborate that exercising and breathing exercises (including abdominal diaphragmatic breathing, semi-closed lips, and increased expiratory time) effectively improve exercise tolerance, endurance, and overall fitness. The result of performing respiratory muscle training lead to increased strength and endurance of the inspiratory muscles, a decrease in respiratory symptoms, and the control of respiratory symptoms as a non-drug therapy, thus promoting patients’ functional capacity. By increasing functional capacity, patients can perform their daily activities more easily and enjoy a better quality of life. Studies have also highlighted the importance of assessing progress in physical capacity and activity tolerance as indicators of increased functional status. Just by regularly tracking and assessing these parameters, healthcare professionals can monitor patients’ progress and adjust interventions as needed.

The outcome indicator *symptom control* involves health education interventions on inhaler therapy [9] and coughing techniques [9,14], breathing control techniques for dyspnea/anxiety relief [8,13,14], and non-pharmacological techniques for symptom control and relief [9,43]. Several studies agree with these findings, suggesting self-care as an outcome sensitive to rehabilitation care, with a positive impact on promoting health and well-being by increasing people’s skills and abilities through aptitude training or adaptive strategies, in which rehabilitation care plays a decisive role [46,47,48,49,50,52]. According to Wang et al. [9], empowering patients to self-manage their disease by providing knowledge about pathophysiology, therapeutic administration techniques, and pharmacological and nonpharmacological strategies for symptom relief reduces patients’ recurring visits to emergency departments during symptom exacerbation because patients and their families are equipped with disease control and management expertise. This indicator is mainly related to the health literacy of patients and their families. Other studies report that it is necessary to include the ability to identify nonpharmacological strategies for pain relief during symptomatic states [51] and to teach strategies to prevent complications during disease exacerbation [18].

Concerning the outcome indicator *self-care capacity*, an inherent relationship was observed with the outcome indicator functional status. Interventions related to functional status also improved self-care ability, and specific interventions focused on self-care also impacted functional status. The various studies reviewed included interventions such as teaching energy conservation techniques [9,14], teaching gait training [14], adaptive strategies with technical aids [9], sensory–motor training [9], and basic life activity training [9,43]. López-Liria et al. [18] reached similar conclusions, corroborating the findings of previous studies. More specifically, these authors rated the interventions toward self-care promotion. Their findings revealed a substantial increase in Barthel Index scores after the implementation of respiratory rehabilitation, indicating a reduction in patient dependence. Patients with chronic respiratory disorders had the best results in increasing functional capacity after respiratory rehabilitation, including interventions such as functional exercises, gait training, and self-care skill training for caregivers.

Two outcome indicators identified in this study were *health-related quality of life* and *patient satisfaction*, which have been shown to be related to all the other indicators [13]. This fact is evidenced by Wang et al. [9], Chung et al. [8], López-Liria et al. [18], Collins et al. [43], and Tsang et al. [14], who showed that RNIs resulted in increased quality of life and satisfaction. According to Collins et al. [43], respiratory rehabilitation decreases the sensation of dyspnea and improves the perception of control over the disease, improving quality of life. These results were corroborated by Tsang et al. [14], in which patients’ quality of life increased after respiratory rehabilitation, reducing psychological symptoms such as anxiety and depression. Moreover, a study conducted in Portugal, which presented a measure of patient satisfaction sensitive to nursing care in older adults, assessing the quality of nursing care provided to this specific population, relates the outcome indicators “functional capacity” and “quality of life” as determinants that influence patient satisfaction [53]. By integrating patient feedback and addressing the factors that impact patient satisfaction, healthcare professionals can improve the quality of nursing care provided to older adults with chronic conditions, resulting in better outcomes and a more positive health experience [54,55].

In the *health promotion activities* outcome indicator, the study developed by Wang et al. [9] presents the largest number of interventions related to this indicator. Also, according to this author, nurse-led self-management through educational programs has been shown to reduce disease exacerbation in COPD patients significantly. These programs empower patients with the necessary skills to manage symptoms and the psychological consequences of the disease, thus reducing the recurrence of emergency services [52]. Tsang et al. [14] also highlight the importance of patient and family education in pulmonary rehabilitation. The participation of family members in health education sessions has shown a direct relationship with increased patient knowledge about the disease [9]. Thus, the inclusion of the family in the care process and their support result in greater health outcomes, including knowledge of the pathophysiology of the disease, disease management, and a reduction in psychological symptoms. According to the World Health Organization (WHO) [21,56], nurses play a crucial role in health promotion through support, education, and instruction for self-care. This involves identifying the needs of patients/families, providing education to bridge knowledge gaps, empowering patients to manage their health, and providing psychological support to promote healthy behaviors. In all five studies eligible for this SLR-MA and the guidelines issued by the WHO, the importance of self-care is clear for developing knowledge, skills, and attitudes that promote the rehabilitation, readaptation, and the reintegration of self-care skills, contributing to the well-being of people with chronic diseases.

Concerning the outcome indicator *healthcare utilization*, it was addressed in two specific studies. In Wang et al. [9], the authors observed that the group undergoing respiratory rehabilitation presented significantly reduced recurring visits to the emergency room compared with the control group. This finding suggests that patients involved in respiratory rehabilitation programs had a reduced need to seek emergency care during the 6-to-12-month intervention period. This reduction may be related to empowering patients in their disease self-management, thus decreasing disease exacerbation. Multidisciplinary interventions and referencing were emphasized to optimize patients’ health. Nurses provide individualized care, considering the specific needs of patients and their families, while working in interdisciplinary coordination. According to Wang et al. [9] and López-Liria et al. [18], the implementation of respiratory rehabilitation programs can lead to decreased utilization of healthcare services, such as emergency room visits, due to the empowerment of patients in managing their illnesses and the efficient coordination of healthcare teams in meeting individuals’ needs. Other studies also state that as people age, they become increasingly frail, presenting functional disabilities that, when accompanied by multimorbidity, more easily decompensate; this is a consequence that can result in disabilities, some of them severe, namely those originating from worsening of their health conditions [54,55]. This scenario generally leads older adults to frequently visit the emergency room with acute health problems, including respiratory disorders, impacting self-care [57].

Although not reported in the five articles screened for this SLR-MA, it is essential to refer to Obstructive Sleep Apnea Hypopnea Syndrome (OSAHS), which can cause excessive daytime sleepiness that significantly impacts older adults’ daily lives, leading to various challenges and limitations. This obstruction can cause dyspnea, a sensation of breathlessness, or difficulty breathing, further contributing to the overall burden experienced by individuals with OSAHS. The combination of daytime sleepiness and dyspnea can lead to reduced energy levels, impaired cognitive function, and decreased quality of life in affected individuals [58]. Therefore, the proper diagnosis, treatment, and management of OSAHS are crucial in alleviating these symptoms and improving the overall well-being and functionality of affected older adults. Nurses can play an important role in helping older adults with OSAHS, providing education and patient counseling, continuous positive airway pressure therapy, regular follow-up visits that can help address any issues or side effects associated with treatment, and ongoing support and encouragement, among other essential nursing interventions, all of them always tailored to the individual’s specific needs and preferences [59,60].

Finally, this SLR-MA also had its limitations, which are mainly related to the fact that the selection of articles only included the English and Portuguese languages, thus limiting the scope of the results obtained and, consequently, leading to the loss of significant information from other, potentially important, international research written in other languages. In addition, the review included only studies from the last five years, thus excluding studies published before the specified period, which may generate the possibility of missing necessary background research, historical contexts, and previous developments in the topic at hand; this may result in an incomplete understanding of RNIs for older adults with respiratory disorders, as previous studies may have laid the groundwork for the current research and provided crucial information.

## 5. Conclusions

The significance of evidence-based nursing practices when guiding interventions and treatments for older adults with respiratory disorders was underscored in this SLR-MA. As reported, nurses can successfully promote health and empower older adults to take charge of their well-being through education, support, and psychological assistance, guiding them to manage their health effectively. Furthermore, integrating family involvement and peer support is essential to promoting self-care in respiratory chronic disorders. Finally, it is also essential to mention the nursing-sensitive indicators identified, which show, in several aspects, the effectiveness of RNIs: functional status, symptom control, self-care capacity, healthcare utilization, health-related quality of life, and patient satisfaction.

## Figures and Tables

**Table 1:** Articles identified and their main results.

Authors (Year), Reference, and Title	Objectives	Participants	Interventions/Phenomena of Interest	Results and Conclusions
**Authors**: Chung, Y.; Huang, T.-Y.; Liao, Y.-H.; Kuo, Y.-C. (**2021**) [8] **Title**: 12-Week Inspiratory Muscle Training Improves Respiratory Muscle Strength in Adult Patients with Stable Asthma: A Randomized Controlled Trial	To investigate and compare the effects of a 12-week intervention using breathing exercises or inspiratory muscle training on lung function, respiratory function, muscle strength, and asthma control (primary outcomes) as well as functional capacity and physical activity (secondary outcomes) in older people with asthma.	Sixty patients with asthma were randomly assigned to the following groups: N = 30 in the intervention group with breathing exercise training (BET); N = 30 in the intervention group with inspiratory muscle training (IMT). The program lasted 12 weeks.	The BET group took part in sessions twice a week for 25 min, performing diaphragmatic and nasal breathing, increasing their expiratory time, decreasing their respiratory flow, and regulating their breathing rhythms. They performed exercises to increase the resistance of the rib cage and abdominal and diaphragmatic strength in association with diaphragmatic breathing techniques and semi-closed lips. The IMT group performed 30 dynamic inspiratory exercises twice a day. The intensity of each inspiration was 50–60% of the maximum inspiratory pressure. Patients initiated inspiration from residual volume to maximal lung pressure for at least 1 s.	Both IMT and BET demonstrated comparable beneficial outcomes in enhancing functional capacity and physical activity. IMT effectively improved respiratory muscle strength (RMS), as evidenced by increased maximal inspiratory pressure. The observed maximal inspiratory pressure ranged from 72% to 82%, indicating the need to improve RMS in the target population of older adults. The impact of IMT on asthma demonstrated an improvement in inspiratory muscle strength and endurance, which supports the effectiveness of IMT as an asthma control strategy, reducing disease severity, as a non-drug therapy. In both groups, maximal expiratory pressure increased, as did RMS and functional capacity. BET and IMT induced increases in the 6-min walking test (6MWT), increasing exercise tolerance. The IMT group tended to show a more significant increase in physical activity time than the BET group, thus leading to the conclusion that implementing health education programs and professional guidance to improve RMS improves ventilatory control, which may be associated with confidence in performing physical activity.
**Authors**: Martin-Sanchez, C.; Barbero-Iglesias, Fausto J.; Amor-Esteban, V.; Martin-Nogueras, Ana M. (**2021**) [13] **Title**: Comparison between Two Inspiratory Muscle Training Protocols, Low Loads versus High Loads, in Institutionalized Elderly Women: A Double-Blind Randomized Controlled Trial	To evaluate and compare the effectiveness of 2 protocols with inspiratory muscle training (IMT), one with low intensity and the other with high intensity, to improve respiratory strength, functional capacity, and dyspnea in institutionalized elderly women over 65 years.	Institutionalized elderly women aged 75–95 years with a Barthel Index score of 60 or more, who were able to walk unaided and who understood the purpose and interventions of the study. The participants were randomly distributed into two groups: N = 14 participants in the high-intensity group (IMT HIGH). N = 12 participants in the low-intensity group (IMT LOW)	A pressure transducer assessed muscle strength by measuring maximal inspiratory and expiratory pressure. The 6-min walking test (6MWT) was used to assess functional capacity. Dyspnea was assessed using the Modified Medical Research Council scale. The inspiratory muscle training intervention protocol took place over 8 weeks, using the threshold, with a frequency of 1 session per day, 5 days a week. The participants performed 15 series of 1 min with 1 min of rest between them. The training intensity differed in both groups: 40% of the maximum inspiratory pressure in the IMT HIGH group and 20% in the IMT LOW group.	Respiratory muscle strength (RMS): A significant improvement in maximal inspiratory pressure occurred in the high-intensity group after the intervention compared to the low-intensity group. Maximal expiratory pressure increased significantly after the intervention in both groups, and the improvement achieved was similar between them. Functional Capacity: The distance walked on the 6MWT by both groups increased significantly after the intervention. Dyspnea: Dyspnea significantly improved in the low-intensity group after the intervention. In summary: This study showed that an 8-week protocol with inspiratory muscle training improves respiratory muscle strength and functional capacity in older women. Training with high loads was most effective for improving inspiratory muscle strength, and training with low loads was best for reducing dyspnea. A minimum load of 20% of the maximal inspiratory pressure was sufficient to improve dyspnea, inspiratory strength, expiratory strength, and functional capacity in older women.
**Authors**: Wang, L.H.; Zhao, Y.; Chen, L.Y.; Zhang, L.; Zhang, Y.M. (**2019**) [9] **Title**: The effect of a nurse-led self-management program on outcomes of patients with chronic obstructive pulmonary disease.	To examine the effectiveness of a nurse-led self-management program on outcomes in patients with chronic obstructive pulmonary disease (COPD)	A total of 154 patients were admitted to the Hospital of Zunyi Medical University in Guizhou with COPD, of whom 77 belonged to the control group and the remaining 77 to the intervention group. The participants were randomly assigned to the groups.	A self-management program was divided into 2 phases. In the first (6–7 days before discharge), the needs and fears of each participant were assessed (social support, barriers to self-management, enjoyment of physical activities) and, according to individual needs, 5 to 6 educational sessions were given by specialist nurses, each lasting 45 min. In each session, topics such as what COPD is and its impact, training the respiratory muscles, half-closed lips and diaphragmatic breathing, medication, inhaler use technique, coughing techniques, non-pharmacological strategies for symptom relief, types of physical exercises adapted to COPD, smoking cessation, and long-term oxygen therapy were addressed. The second phase comprised: (i) phone calls every week for 3 months (checking compliance with the self-management program, providing encouragement and positive reinforcement, checking for complications, and assessing exercise for duration and intensity); (ii) 3 home visits (assessing environmental risk factors, encouraging modification of health behaviors, reinforcing knowledge and self-management, providing understanding and emotional support).	The intervention group used the emergency room less often at 6 and 12 months of intervention than the control group, perhaps because patients were empowered to self-manage their disease and received appropriate interventions simultaneously, thus contributing to a reduction in disease exacerbation. Exercise capacity and tolerance levels increased after 3 and 6 months of intervention in the intervention group compared to the control group. Health-related quality of life increased in the intervention group, while its deterioration occurred in the control group. At 6 and 12 months of intervention, the intervention group was more satisfied with the program, service, and education provided than the control group. The findings of this study showcased that introducing a self-management program led by nurses, which focuses on fostering the skills and capabilities required for effectively managing therapy, symptoms, and psychological impacts in patients with COPD, resulted in decreased visits to the emergency department due to disease exacerbation. Additionally, the program demonstrated enhancements in exercise capacity, functional capacity, health-related quality of life, and overall satisfaction with the provided care.
**Authors**: Collins, E.G.; Jelinek, C.; O’Connell, S.; Butler, J.; Reda, D.; Laghi, F. (**2019**) [43] **Title**: The Effect of Breathing Retraining Using Metronome-Based Acoustic Feedback on Exercise Endurance in COPD: A Randomized Trial.	The primary objective of this study was to explore the impact of exercise training with respiratory re-education through acoustic feedback, using a metronome, during exercise in patients with chronic obstructive pulmonary disease (COPD). The secondary objective was to evaluate the impact of respiratory re-education on dynamic hyperinflation and patients’ health-related quality of life.	A total of 119 COPD patients were randomly selected from a population of 205, with 58 in the intervention group and 61 in the control group.	There were 36 training sessions, 3 times a week, in the laboratory. Both groups started by training for 25 min on a treadmill and progressed, according to tolerance, to 45 min. The intervention group was previously instructed to inhale through their noses and exhale with their lips half-closed in time with the metronome sound. The patients in the control group were free to choose how to control their breathing.	Although changes in breathing pattern were obtained in the intervention group, the exercise duration and hyperinflation results were equivalent in both groups. After 12 weeks of the program, the intervention group showed a 9% increase in expiratory time, a 9% decrease in respiratory rate, and 3% dynamic hyperinflation. However, this improvement was minor and insufficient to associate exercise training with respiratory re-education using acoustic feedback with a metronome to increase exercise tolerance compared to exercise training alone. The conclusion of the training program was that both groups witnessed enhancements in dyspnea, fatigue, mental functions, and patient perception of disease control, alongside an increase in health-related quality of life. In both groups, patients reported a significant decrease in dyspnea when performing their activities of daily living, suggesting that changes in breathing patterns in the acoustic feedback training group had no impact on this symptom.
**Authors**: Tsang, E.W.; Kwok, H.; Chan, A.K.Y.; Choo, K.L.; Chan, K.S.; Lau, K.S.; Chan, C.C.H. (**2018**) [14] **Title**: Outcomes of community-based and home-based pulmonary rehabilitation for pneumoconiosis patients: a retrospective study.	To analyze the clinical benefits of pulmonary rehabilitation programs in the community and at home in patients with pneumoconiosis.	From a population of 685 patients with pneumoconiosis enrolled in community pulmonary rehabilitation (CPR) or home pulmonary rehabilitation programs (HPRP) offered by three hospitals in Hong Kong, 155 patients in CPR and 26 patients in HPRP were selected as the study sample. The mean age of the participants in CPR was 70.74 and that of the participants in HPRP was 74.54 years.	Data were grouped into 2 categories according to the following: (i) the demographic characteristics of the patients, specific information about the disease, and the program in which they participated (age, sex, degree of disability, body mass index, forced expiratory volume, and smoking history); (ii) the outcomes used to measure the benefits of pulmonary rehabilitation programs, through various scales, were the Chronic Respiratory Questionnaire (CRQ), Hospital Anxiety and Depression Scale (HADS)], Physical (PCS) and Mental (MCS) Health Scales Short Form-12 (SF-12), knowledge (included as an additional file to the article), and the 6-Min Walk Test (6MWT).	The findings of this study indicate that CPR had a beneficial impact on the patients’ overall well-being, leading to a decrease in psychological issues like anxiety and depression. Moreover, the assistance provided by healthcare practitioners, along with home visits and educational sessions for families, were factors linked to a reduction in depressive symptoms. Additionally, a strong correlation was observed between family involvement in these sessions and the improvement of patients’ understanding of the disease. As a result of participating in the program, patients experienced enhanced mobility and increased exercise capacity. Both pulmonary rehabilitation programs involved physical and respiratory training, increasing the patients’ levels of exercise tolerance. The effectiveness of the training period was found to be greater when it was extended, leading to improved physical capacity. During the hygiene education sessions, patients were educated on respiratory hygiene techniques, inhaler usage, and energy conservation methods. The outcomes indicated that patients enhanced their ability to manage their health and achieved higher levels of exercise tolerance.

**Table 2:** Summary of the meta-analysis results of the individual studies included in the present SLR-MA that were found to be eligible for analysis upon standardizing the means between the control and intervention groups regarding the 6MWT variable.

Study Name	Std Diff in Means ^1^	Standard Error	Variance	Lower Limit ^2^	Upper Limit ^3^	*Z*-Value	*p*-Value
Chung et al. [8]—BTE	0.339	0.260	0.068	−0.170	0.849	1.305	0.192
Chung et al. [8]—IMT	0.548	0.263	0.069	0.033	1.064	2.085	0.037
Martin-Sanchez et al. [13]—IMT High	0.199	0.379	0.144	−0.544	0.941	0.525	0.600
Martin-Sanchez et al. [13]—IMT Low	0.324	0.411	0.169	−0.482	1.129	0.787	0.431
Wang et al. [9]	1.321	0.178	0.032	0.972	1.669	7.425	<0.001
Tsang et al. [14]—CBRP	0.658	0.108	0.012	0.447	0.870	6.097	<0.001
Tsang et al. [14]—HBRP	0.423	0.213	0.045	0.005	0.840	1.983	0.047
Fixed-effect model	0.682	0.074	0.006	0.536	0.827	9.186	<0.001

^1^ Standardized difference in means, which is the effect size measure used to compare means between the control and intervention groups; ^2^ lower bound of the 95% confidence interval of the mean; ^3^ upper bound of the 95% confidence interval of the mean.

## Data Availability

All data and materials in this research can be obtained by contacting the author Rita Ribeiro (ritiribeiro@hotmail.com).

## Appendix A

**Table A1:** Patient outcome indicator framework used to systematize the nursing interventions.

Domains	Rehabilitation Nursing Interventions
Functional status	Training sessions for the respiratory muscles (abdominal diaphyseal breathing, half-closed lips, increased expiratory time) [8,14,43]; Exercise training [9,14,43]; Assessment of progress in physical capacity/activity tolerance [8,9,13,14,43]; Assessment of lung function [8,13,14,43]; Functional capacity assessment [8,13,14]; Use of threshold for inspiratory muscle training [13]; Promoting functioning [9,13,14,18,43,51].
Symptom control	Teaching inhaler therapy technique [9]; Teaching effective coughing technique [9]; Teaching non-pharmacological techniques for symptom control and relief [9,14,43]; Assessment of dyspnea/anxiety [13,14]; Teaching breathing control techniques (muscle relaxation and dissociation of breathing times) for dyspnea/anxiety relief [13,14].
Self-care capacity	Teaching energy conservation techniques [9,14]; Teaching and training gait [13]; Training basic life activities [43].
Health-related quality of life	Assessment of the increase in health-related quality of life [8,9,14,43].
Health promotion activities	Identifying needs in terms of disease self-management and caregiver/family management [9]; Conducting health education sessions aimed at empowering the person and disease self-management [9,14]; Monitoring/assistance through structured follow-up system in order to guide health-seeking behavior [8,9]; Conducting smoking cessation sessions [9]; Providing psychological support/providing psychosocial support [9,14]; Promoting the inclusion of the family in the care process and their support [9,14].
Healthcare utilization	Monitoring of the rate of recurring visits/returns to emergency services [9].
Patient satisfaction	Assessing the level of patient/family satisfaction with care [9].
